# Hysteria and Crime Mimicry

**Published:** 1904-10

**Authors:** 


### HYSTERIA AND CRIME MIMICRY.

"When you see a paragraph," remarks Dr. Wilks (Lectures on Diseases of the Nervous System), "headed 'extraordinary occurrence,' and you read how every night loud rapping is heard in some part of the house, or how the rooms are being constantly set on fire, or how all the sheets in the house are being devoured by rats, you may be quite sure there is a young girl on the premises." "There are," remarks Ross (Diseases of the Nervous System), "few depths of human degradation which hysteriacs fail to reach. They sometimes intrigue against their friends, and maintain that they are persecuted and outraged, and play their part with such consummate skill as to deceive physicians and judges. Girls about puberty sometimes commit the greatest crimes which it is possible to imagine, without recognizable motive and apparently from sheer love of mischief. A young lady at a boarding-school has torn her sheets and underclothing into shreds, and then endeavored to fasten the guilt upon a schoolmate. It is not unusual for the psychic disturbances of hysteria to assume an erotic character. Girls may then assert that they have been ravished, and usually maintain that the most outrageous violence was used by the perpetrator of the crime, or that they themselves were previously drugged."

The *British Medical Journal* about two decades ago (December 11, 1884), anent the alleged assault on Lady Florence Dixie by Fenians, "directed attention to certain remarkable delusions to which females of unstable nervous equilibrium are subject, either through hysteria or through similar disorders of the nervous system. Charcot and Bourneville give instances of the extraordinary self-deceptions that are frequent among hysteriacs." Legrand du Saulle describes (Les Hysteriques) cases where females labor under the belief that they had been struck or stabbed by others even after having inflicted blows and wounds upon themselves. In one instance a young woman was found by her husband lying on the floor of her room in a fainting fit, her face covered with blood. On reviving from her swoon she stated that she had been attacked by armed men. The Paris newspapers dilated on the case, and within three weeks two similar events occurred in Paris. All these cases proved to be fabricated by the supposed victim. A young woman wounded herself slightly with a pistol. She gave the police authorities the most minute details about an imaginary assassin who inflicted the wound. She was found to be markedly hysterical and to have wilfully wounded herself. A young woman was found in a railway carriage stabbed in the left side. The incident caused great excitement, but it was proved that she had inflicted the wound herself and was a hysteriac. A housemaid, who was found lying behind the door, bound, gagged, and covered with bruises, stated that she had been brutally attacked by two burglars with blackened faces. She was a decided hysteriac, and had contrived to tie her own hands, gag and bruise herself. In a case reported by Tardieu, a young woman wished to make herself an object of public interest by passing as the victim of a political conspiracy which she claimed to have discovered. One night she was found in great mental perturbation at the door of her apartment. She could not talk, but wrote that she had been attacked outside her house by a man who had attempted to garrote her, at the same time striking her twice with a dagger. Only her clothing was injured. The body of her dress and her corset were cut through at different levels. She claimed that the attempt at strangulation had caused dumbness. Tardieu remarked in her hearing (Etude Médico-Légale sur la Folie) that this infirmity rapidly disappeared when produced under such circumstance. She soon regained her speech, and in a short time admitted that the whole narrative had been evolved out of her inner consciousness.

In a case reported by Toulmouchs (*Ann d'Hyg. Pub.*, S. I, T. I, 1853) a young girl was given to devotional exercises and much inclined to flagellation and asceticism. One day she cut herself with scissors six hundred times on various parts of the body. She asserted these wounds were made by a man who tried to outrage her, but finally confessed that the injuries were all self-inflicted. In a case cited by Tardieu, a young girl charged two men with having violated her and introduced into her rectum and vagina stones, pieces of wood, and iron, which had to be extracted with great pain. She had convulsive seizures followed by paresis. The two men were convicted and had been imprisoned for more than a year when the false nature of the accusation was discovered. In the Bond case, which occurred about twenty years ago in Illinois, J. G. Kiernan points out (*Journal of Nervous and Mental Diseases*, January, 1885) that the first accusation made was vague and indefinite, and that only as suspicion pointed to certain persons did the charges of Miss Bond become direct and positive. Her testimony as a whole was so contradictory that the jury threw it out of consideration. The prosecution failed to place the physicians who examined Miss Bond, after the alleged outrage, upon the stand. The defense claimed that they had the right to cross-examine these physicians, but this claim was not allowed. There were insane and eccentric members in the Bond family. During the period between the time of the alleged outrage and the trial, whenever public interest in the case began to flag, Miss Bond had hysterical convulsions and was on the point of death so frequently that the newspapers commented on her tenacity of life. She had herself photographed and the photograph sold to defray the cost of extra counsel for the prosecution. Her only claim to public notice was the fact that she was outraged. Taking into account the indefinite, contradictory nature of the earlier accusations, the craving for public notice shown in the sale of the photographs, the convenient convulsive attacks at periods when interest was abating, insignificant non-use of medical testimony by the prosecution, the existence of neurotic relatives, but one conclusion could be drawn: the charge was a hysteric product which imposed on a scandal-loving community. The jury found the prisoners innocent in the face of public clamor for conviction.

In a Manchester, England, case reported by Cullingworth [the medical witness called by the police to investigate the case after supposed dying statements had been taken (Ross Diseases of the

Nervous System)] a girl of eighteen was found, with her clothing wet and muddy, in an apparently stupefied condition in the closed doorway of a restaurant, a few yards from her lodging. She was taken home and to bed, and a physician was sent for, who found her seemingly unconscious of what was going on around her, uttering some disjointed and incoherent complaints of having been drugged and threatened. He thought she was recovering from a narcotic, and did not at first pay any attention to her story. The following morning she appeared worse. In the evening her condition was considered so critical that the police were notified. She was visited by two detectives, who, on the physician's assurance that she was in a dying state, sent at once for a magistrate, before whom she made a solemn declaration to the effect that she believed herself to be dying, and that on the previous evening a solicitor, at whose office she had called on business, told her that she must go into a convent, and gave her "some sort of dark, sweet drink," which rendered her senseless. On going down-stairs from the office she met a Jesuit, whom she had seen once before. He seized her and pulled her along the street to a little house in a court, where there was an upper room with a bed in it and a cross on the wall. Here he said improper things to her, and gave her a little cake which affected her directly. The woman of the house came into the room and found her on the floor, after which she somehow got outside, the priest following. He again dragged her along in the dirt to the street corner, when he ran away. The story was proven purely imaginary, and the case was dismissed, after the solicitor and the priest, both highly respected, had been placed under arrest in the middle of the night.

Cook county, as was well remarked by Lieutenant Heidelmeier of the Chicago police, has lately been having an epidemic of girls of the hysteric type. Gertrude Spoden, a sixteen-year-old girl, fell exhausted at the door of a house under much the same circumstances as the Manchester case just cited. She told a story of seizure by an elderly man who dragged her into a cab, and there with a companion tortured her with burning matches and hat-pins. Later she confessed that the story was a fabrication and that she had inflicted the wounds herself in a public park. In an Evanston case a nervous woman, who had long been under treatment and had repeatedly been operated on for various nerve disturbances, received about three years ago great commendation as a heroine for repelling burglars, no traces of whom were discoverable. She was later, according to her story, visited by burglars who threatened to throw carbolic acid in her face if she screamed, which she did, whereupon the acid was thrown. A bottle was found containing a very weak, harmless solution of carbolic acid. Some property was scattered through the house in the style of the burglar imagined by an inexperienced person. The police were unable to discover any traces of burglary and abandoned the case. The physician who accepted the story of Gertrude Spoden claimed that she could not stand the pain of such wounds long enough to inflict them. That this is a common error of neurologically untrained physicians is shown in their frequent testimony as to the signficance of the pain test in accident cases, where even maligners are able to hypnotize themselves into analgesia. The same phenomenon appears in the "horses" or trained subjects of professional hypnotists. One of these some years ago ran needles through his arms to gather crowds, whose pockets his accomplices rifled. The sensation tests used by physicians in "miracle" and accident cases are tremendously fallacious and misleading. In hysteric crime mimicry they become sociologically dangerous through supporting false accusations.—*Medicine.*

